# Association of serum selectins with outcomes in older adults following hip fracture surgery: a 3-year follow-up study

**DOI:** 10.3389/fmed.2026.1932543

**Published:** 2026-10-27

**Authors:** Shengyu Wang, Sen Li, Da Chen, Feng Gao, Xiaofei Song

**Affiliations:** Department of Joint Surgery, Nanyang Central Hospital, Nanyang, Henan, China

**Keywords:** E-selectin, hip fracture, L-selectin, older adults, outcome, P-selectin

## Abstract

**Background:**

Hip fractures represent a critical public health challenge for older adults, often leading to severe mobility decline and high long-term mortality. Selectins (E-, L-, and P-selectin) are cell adhesion molecules that play complementary roles in inflammation, hematopoiesis, and bone remodeling; however, their prognostic value in orthopedic trauma remains largely unexplored.

**Methods:**

A 3-year, single-center, observational cohort study was conducted involving patients older than 60 years who underwent surgical intervention for primary, low-energy hip fractures between June 2019 and June 2022. Fasting peripheral venous blood samples were collected on the first morning after admission to quantify serum selectin levels using ELISA. Multivariable Cox and Logistic regression in the full cohort served as the primary analysis, complemented by a 1:1 propensity-score-matched case-control comparison, Receiver Operating Characteristic (ROC) curve analysis for risk stratification, and Kaplan–Meier survival curves.

**Results:**

The final analysis included 431 patients, comprising 302 patients who survived >3 years and 129 who survived ≤3 years. In the fully adjusted multivariable model, above-cutoff P-selectin was an independent predictor of 3-year mortality (HR = 3.167, 95% CI: 2.170–4.622; *p* < 0.001). In a 1:1 propensity-score-matched case-control subset (*n* = 232; 116 pairs), patients who died within 3 years exhibited significantly higher preoperative P-selectin levels than survivors (median 4.18 [IQR 0.98–18.21] vs. 1.32 [0.43–7.51] ng/mL; *p* < 0.001). Using the optimal ROC cutoffs, the above-cutoff P-selectin group demonstrated a markedly elevated 3-year mortality rate (58.67% vs. 23.88%; RR = 2.46, 95% CI: 1.88–3.20; *p* < 0.001) and a significantly lower 3-year independent ambulation rate (38.67% vs. 65.73%; RR = 0.59, 95% CI: 0.44–0.79; *p* < 0.001) compared with the below-cutoff group. Patients with above-cutoff E-selectin also experienced a significantly higher 3-year mortality rate (38.13% vs. 26.03%; RR = 1.46, 95% CI: 1.10–1.95; *p* = 0.01). In multivariable analyses of the full cohort, above-cutoff P-selectin was an independent predictor of both mortality (HR = 3.167; *p* < 0.001) and a decreased likelihood of independent ambulation (OR = 0.346; *p* < 0.001). Above-cutoff E-selectin was associated with mortality in the univariable analysis (HR = 1.569; *p* = 0.012), but this association was attenuated and no longer statistically significant after multivariable adjustment (HR = 1.397; *p* = 0.072). L-selectin did not show a statistically significant impact on any clinical endpoint.

**Conclusions:**

Elevated preoperative serum P-selectin is an independent predictor of long-term mortality in older adults following hip fracture surgery. P-selectin may also serve as a potential prognostic indicator for poor functional recovery and a failure to regain independent ambulation, although this association was attenuated among 3-year survivors and should be interpreted in the context of the competing risk of death. The univariable association between E-selectin and mortality did not persist after multivariable adjustment and requires confirmation in larger cohorts.

## Introduction

Hip fractures represent a critical public health challenge among older adults, characterized by high morbidity, a significant loss of functional independence, and elevated long-term mortality (1–5). Following hip fracture surgery, many older patients experience a profound decline in mobility and overall quality of life (6, 7). Consequently, identifying reliable prognostic factors is essential for clinical risk stratification (6, 8, 9). Exploring novel biomarkers could provide valuable insights into the biological mechanisms associated with postoperative recovery, helping clinicians predict long-term outcomes, such as mortality and independent ambulation, to better tailor early interventions and rehabilitation strategies.

Selectins are a family of calcium-dependent, transmembrane cell adhesion molecules that play distinct and complementary roles in inflammation, hematopoiesis, and immune responses (10–13). The selectin family comprises three main types: E-selectin, L-selectin, and P-selectin. E-selectin is exclusively expressed on endothelial cells (14, 15). P-selectin is prestored in the α-granules of platelets and the Weibel-Palade bodies of endothelial cells (16, 17). L-selectin is primarily expressed on circulating leukocytes (11, 18). Together, these molecules are fundamentally responsible for mediating the initial tethering and rolling of leukocytes along the vascular endothelium during inflammatory cascades (19–22).

Emerging evidence suggests that selectins may have a significant prognostic role in older adults with hip fractures, primarily driven by two potential mechanisms. First, selectins and their corresponding ligands can directly influence bone metabolism. The systemic inflammatory response triggered by surgical intervention in osteoporotic fractures is accompanied by endothelial activation and altered circulating selectin concentrations (23). Moreover, selectin ligands such as E-selectin ligand-1 act as critical regulators of bone remodelling by limiting bioactive transforming growth factor-beta in the bone microenvironment (24). Second, high concentrations of P-selectin have been positively correlated with the incidence of sarcopenia (25). Because sarcopenia inherently impairs muscle mass, physical performance, and postural stability, elevated selectin levels may be strongly associated with the poor functional recovery and worse overall prognosis commonly observed in patients after a hip fracture (26, 27).

While the roles of selectins in inflammation and bone remodeling are increasingly recognized, their clinical relevance as prognostic indicators in orthopedic trauma remains unexplored. We hypothesized that altered serum concentrations of E-, L-, and P-selectin would reflect the underlying inflammatory and metabolic derangements following hip fracture, thereby predicting poorer survival and functional recovery. To test this, we conducted a 3-year, single-center follow-up study to evaluate the association of these three selectins with 3-year all-cause mortality and the restoration of independent ambulation in older adults undergoing hip fracture surgery.

## Methods

### Study design and participants

This single-center, prospective observational study was conducted at the Department of Joint Surgery, Nanyang Central Hospital, Nanyang, Henan, China. Consecutive eligible patients were enrolled between June 2019 and June 2022; peripheral venous blood was collected on the morning after admission and banked at −80 °C for pre-planned biomarker measurement, and clinical variables were extracted from the hospital's electronic medical records system. The study protocol was approved by the Institutional Ethics Committee of Nanyang Central Hospital (approval number: [20180782]) and adhered strictly to the ethical principles of the Declaration of Helsinki. Because this was a non-interventional observational cohort study it was not prospectively registered in a public trial registry, and the manuscript was prepared in accordance with the STROBE statement for observational studies. Written informed consent was obtained from all participants or their legally authorized representatives prior to enrollment.

We screened consecutive older adults who underwent surgical intervention for primary hip fractures between June 2019 and June 2022. The inclusion criteria were as follows: (1) age >60 years; (2) diagnosis of a low-energy, primary hip fracture (femoral neck or intertrochanteric fracture); and (3) surgical treatment. Patients were excluded if they met any of the following criteria: (1) pathological fractures secondary to malignancy or metabolic bone diseases; (2) concomitant severe multiple traumas or polytrauma (patients with minor superficial injuries were not excluded); (3) non-surgical (conservative) management; (4) incomplete clinical records or lost to the 3-year follow-up.

### Data collection and clinical variables

Baseline demographic and clinical characteristics were systematically extracted from the hospital's electronic medical records system. Collected data included age, sex, body mass index (BMI), fracture classification, time from injury to surgery, and lifestyle factors (smoking and alcohol consumption). The variable recorded as “polytrauma” in the source registry denotes minor concomitant injuries (for example superficial skin abrasions or minor soft-tissue contusions) and does not include severe multiple trauma, which was an exclusion criterion. To accurately quantify the burden of preoperative comorbidities, the Charlson Comorbidity Index (CCI) was calculated for each patient (28). Additionally, routine preoperative laboratory parameters, including red blood cell (RBC) count, hemoglobin (Hb), fasting blood glucose (GLU), serum albumin (ALB), platelet count (PLT), and C-reactive protein (CRP) levels, were retrieved from the central laboratory database.

### Quantification of serum selectins (ELISA)

Fasting peripheral venous blood samples (5 mL) were collected from all eligible participants on the first morning after admission. The whole blood was allowed to clot at room temperature for 30 min before centrifugation at 3,000 rpm for 15 min. The isolated serum was carefully aliquoted and stored at −80 °C until further analysis to prevent protein degradation.

The serum concentrations of E-selectin, L-selectin, and P-selectin were quantified utilizing commercially available human Enzyme-Linked Immunosorbent Assay (ELISA) kits (E-selectin: Ab303747 Abcam; L-selectin: Ab314373 Abcam; P-selectin: Ab100631 Abcam). All procedures were performed in strict accordance with the manufacturer's instructions. Briefly, diluted serum samples and standards were added to microplate wells pre-coated with highly specific capture antibodies. Following adequate incubation and thorough washing, the respective detection systems were applied according to each kit's protocol: for E-selectin and L-selectin, horseradish peroxidase (HRP)-conjugated detection antibodies were used; for P-selectin, a biotinylated detection antibody followed by HRP-streptavidin was added. A colorimetric substrate solution was then introduced, and the enzymatic reaction was terminated with stop solution. Optical density (OD) was measured at 450 nm using a microplate reader. Target selectin concentrations were interpolated from standard curves generated in each assay. For all analyses, serum samples were diluted prior to measurement as recommended by the manufacturer (1:50 for E-selectin, 1:20 for L-selectin, and 1:50–1:500 for P-selectin). The standard curves demonstrated excellent linearity within the validated detection ranges for E-selectin (15.63–1,000 pg/mL), L-selectin (1.25–40 ng/mL), and P-selectin (0.041–30 ng/mL). All samples were run in duplicate using a single kit lot by operators blinded to clinical outcomes, with both intra- and inter-assay coefficients of variation consistently <12% across all assays. Samples were stored continuously at −80 °C without freeze-thaw cycles, and a *post-hoc* analysis confirmed no significant correlation between storage duration and measured selectin concentrations (all *p* > 0.05).

### Follow-up and clinical outcomes

All participants were prospectively tracked for a full duration of 3 years (36 months) post-surgery. Follow-up evaluations were conducted at 3, 6, 12, 24, and 36 months via scheduled outpatient clinic visits or standardized telephone interviews conducted by trained clinical staff.

The primary outcome of the study was 3-year all-cause mortality, defined as the time interval from the date of surgical intervention to the date of death from any cause. Patients who remained alive at the end of the 3-year follow-up period were treated as censored observations. The secondary outcome was the rate of independent ambulation at the 3-year mark. Independent ambulation was clinically defined as the patient's ability to perform routine daily walking activities and mobilize indoors and outdoors without the physical assistance, accompaniment, or supervision of another person (the use of a single cane or walker was permitted).

### Statistical analysis

Continuous variables were assessed for normality using the Shapiro–Wilk test. Variables that were not normally distributed are presented as median [interquartile range] and compared using the Mann–Whitney *U* test, whereas the few normally distributed variables are presented as mean ± standard deviation (SD) and compared using the independent-samples Student's *t*-test; the statistic used is indicated for each variable in the corresponding table. Categorical variables were reported as frequencies with percentages and compared via the Chi-square test or Fisher's exact test, as appropriate.

While multivariable regression on the full cohort served as the primary analysis, a 1:1 Propensity Score Matching (PSM) was implemented to rigorously isolate the association between biomarkers and mortality. Matching was performed using a nearest-neighbor algorithm without replacement, based on propensity scores estimated via logistic regression. A caliper width of 0.2 of the pooled standard deviation of the logit of the propensity score was applied. The comprehensive matching model included the following baseline covariates: age, sex, BMI, smoking and alcoholism history, electrocardiogram and chest radiograph abnormalities, hypertension, fracture history, CCI score, fracture location, surgical procedures, anesthesia type, minor concomitant injuries, time from injury to surgery, RBC, Hb, ALB, GLU, PLT, and CRP. Biomarkers (E-, L-, and P-selectin) were strictly excluded from the matching model. Covariate balance was assessed using Standardized Mean Differences (SMD).

Receiver Operating Characteristic (ROC) curves were generated to evaluate the predictive performance of E-, L-, and P-selectin concentrations for the outcomes, and pairwise comparisons between markers were performed using the DeLong test. Because the survival endpoints are subject to censoring, time-dependent ROC analyses were additionally performed at 3, 12 and 36 months. The optimal cutoff value for each biomarker was determined from the ROC curve for 3-year overall survival in the full cohort by maximising the Youden index. Because these cutoffs were derived and applied within the same dataset, the continuous-variable models were prespecified as the primary analyses and the dichotomised analyses as secondary. Based on these cutoffs, patients were stratified into above-cutoff and below-cutoff subgroups for further comparative analysis. Survival probabilities were estimated using the Kaplan–Meier method, and differences between selectin subgroups were assessed via the log-rank test. Furthermore, multivariable Cox proportional hazards regression models were constructed to evaluate the independent predictive value of selectins on 3-year mortality, while multivariable logistic regression models were utilised to identify independent predictors of independent ambulation.

A two-sided *p* value of <0.05 was considered statistically significant. Because the analyses involved three biomarkers, two parameterisations, two outcomes and three time points, all results other than the primary adjusted P-selectin analyses should be regarded as exploratory and are reported without formal correction for multiplicity. All statistical analyses and graphical visualisations were performed using R software version 4.3.0 (R Foundation for Statistical Computing, Vienna, Austria) with the MatchIt, survival, survminer, timeROC, pROC and rms packages.

## Results

### Study population and baseline characteristics

A total of 761 patients with hip fractures admitted to our department between June 2019 and June 2022 were initially screened. Based on predefined criteria, patients were excluded from the study for the following reasons: age <60 years (*n* = 103), high-energy fractures (*n* = 18), pathological fractures (*n* = 9), no surgical procedures conducted (*n* = 62), and unavailable data, defined as the absence of a valid serum selectin measurement (*n* = 96). Following these exclusions, 473 individuals were enrolled for the measurement of serum selectins. During the follow-up period, 42 patients were lost to follow-up. Ultimately, 431 individuals were enrolled in the final analysis to evaluate the relationships between serum selectins and clinical outcomes (Figure 1).

**Figure 1:**
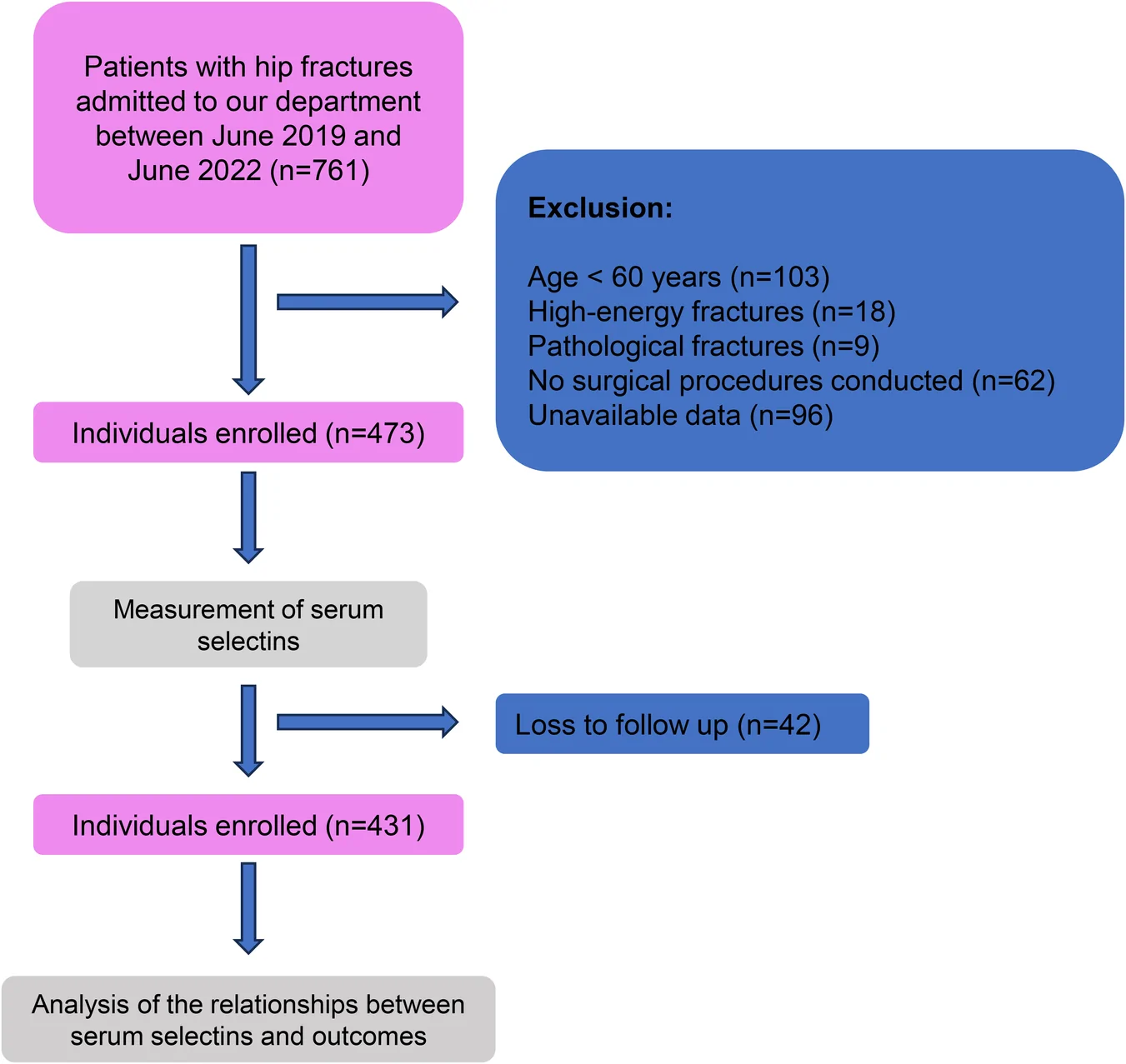
Flow diagram of the study participant selection process.

Prior to propensity score matching (PSM), the unmatched cohort comprised 431 patients, including 302 patients with a survival time of >3 year and 129 patients with a survival time of ≤3 year (Table 1). Baseline comparisons revealed significant differences in age and P-selectin levels between the two groups. Specifically, compared to patients surviving >3 year, those with a survival time of ≤3 year were significantly older (median 77.00 [IQR 71.00–82.00] vs. 71.00 [63.25–77.00] years, *p* < 0.001) and exhibited significantly higher serum P-selectin levels (median 3.75 [IQR 0.93–17.85] vs. 1.77 [0.46–5.51] ng/mL, *p* < 0.001). No statistically significant differences were observed in the remaining baseline demographic and clinical characteristics, including body mass index (BMI), sex, smoking or alcoholism history, electrocardiogram or chest radiograph abnormalities, hypertension, Charlson Comorbidity Index (CCI), fracture location, surgical procedures, anesthesia type, time from injury to surgery, laboratory parameters (RBC, Hb, ALB, GLU, PLT, CRP), and the other selectins (E-selectin and L-selectin) (all *p* > 0.05).

**Table 1:** Baseline demographic, clinical, and laboratory characteristics of the study cohort before 1:1 propensity score matching.

Variables	Unmatched populations (*n* = 431)			*p* value
	Overall	Survival > 3 year	Survival ≤ 3 year	
	(*n* = 431)	(*n* = 302)	(*n* = 129)	
Age (years)	72.00 [66.00, 79.50]	71.00 [63.25, 77.00]	77.00 [71.00, 82.00]	<0.001
BMI (kg/m^2^)	22.66 [19.72, 25.91]	22.30 [19.66, 25.71]	23.07 [19.93, 26.17]	0.144
Sex (female)	299 (69.37%)	204 (67.55%)	95 (73.64%)	0.209
Smoking history (yes)	44 (10.21%)	30 (9.93%)	14 (10.85%)	0.773
Alcoholism history (yes)	29 (6.73%)	19 (6.29%)	10 (7.75%)	0.579
Electrocardiogram (abnormal)	245 (56.84%)	168 (55.63%)	77 (59.69%)	0.436
Chest radiograph (abnormal)	220 (51.04%)	151 (50.00%)	69 (53.49%)	0.507
Hypertension (yes)	260 (60.32%)	177 (58.61%)	83 (64.34%)	0.265
Location of fracture (femoral neck)	250 (58.00%)	178 (58.94%)	72 (55.81%)	0.547
Minor concomitant injuries (yes)	67 (15.55%)	48 (15.89%)	19 (14.73%)	0.76
Fractures history (yes)	72 (16.71%)	50 (16.56%)	22 (17.05%)	0.899
Surgical procedures (arthroplasty)	197 (45.71%)	134 (44.37%)	63 (48.84%)	0.394
Anesthesia (spinal)	4 (0.93%)	2 (0.66%)	2 (1.55%)	0.74
Time from injury to surgery (days)	5.00 [5.00, 5.00]	5.00 [5.00, 5.00]	5.00 [5.00, 5.00]	0.062
RBC (10^12^/L)	4.37 [3.82, 4.87]	4.40 [3.82, 4.86]	4.30 [3.82, 4.89]	0.754
Hb (g/L)	97.78 [89.03, 106.03]	97.60 [89.59, 105.99]	97.87 [87.80, 106.06]	0.781
ALB (g/L)	37.94 [32.95, 43.23]	37.84 [32.82, 43.81]	38.59 [33.49, 42.92]	0.675
GLU (mmol/L)	5.91 [5.13, 6.68]	5.94 [5.13, 6.65]	5.71 [5.13, 6.74]	0.752
CRP (mg/L)	2.88 [1.51, 7.04]	2.84 [1.49, 6.54]	3.07 [1.58, 8.79]	0.32
PLT (10^9^/L)	230.00 [180.50, 284.00]	232.00 [184.00, 292.75]	220.00 [173.00, 263.00]	0.1
CCI score (scores)	3.00 [1.00, 5.00]	3.00 [1.00, 5.00]	3.00 [1.00, 6.00]	0.553
E-selectin (pg/mL)	43.98 [26.42, 438.68]	42.07 [25.82, 427.05]	385.54 [27.37, 474.50]	0.074
L-selectin (ng/mL)	17.88 [12.17, 22.08]	18.25 [12.37, 22.37]	17.12 [11.36, 21.98]	0.406
P-selectin (ng/mL)	2.28 [0.50, 14.28]	1.77 [0.46, 5.51]	3.75 [0.93, 17.85]	<0.001

To isolate the univariable association of the biomarkers with mortality under balanced baseline conditions, a 1:1 propensity score matching analysis was conducted as a secondary analysis. Following matching, 232 patients were successfully paired (116 in the survival >3 year group and 116 in the survival ≤3 year group). As detailed in Supplementary Table S1 and S2, the matching process substantially improved covariate balance: all absolute Standardized Mean Differences (SMD) were reduced to below 0.15 (Supplementary Figure S1). The largest residual imbalances were observed for serum albumin (|SMD| = 0.148), body mass index (|SMD| = 0.139) and hemoglobin (|SMD| = 0.135); all other covariates achieved |SMD| < 0.09 and every covariate remained well below the conventional threshold of 0.25. Balance was markedly better than before matching (for example, the |SMD| for age decreased from 0.713 to 0.021). Notably, even within this balanced matched cohort, P-selectin levels remained significantly higher in the survival ≤3 year group than in the survival >3 year group (median 4.18 [IQR 0.98–18.21] vs. 1.32 [0.43–7.51] ng/mL, *p* < 0.001). These findings in the matched case-control subset are consistent with the primary full-cohort multivariable analysis and reinforce the association between elevated admission P-selectin levels and poorer survival.

### Receiver operating characteristic (ROC) curve analysis and risk stratification

To evaluate the prognostic utility of the serum selectins, Receiver Operating Characteristic (ROC) curve analysis was performed at three follow-up time points (3 months, 12 months, and 3 years) to predict both survival and independent ambulation in the full unmatched cohort (Figure 2).

**Figure 2:**
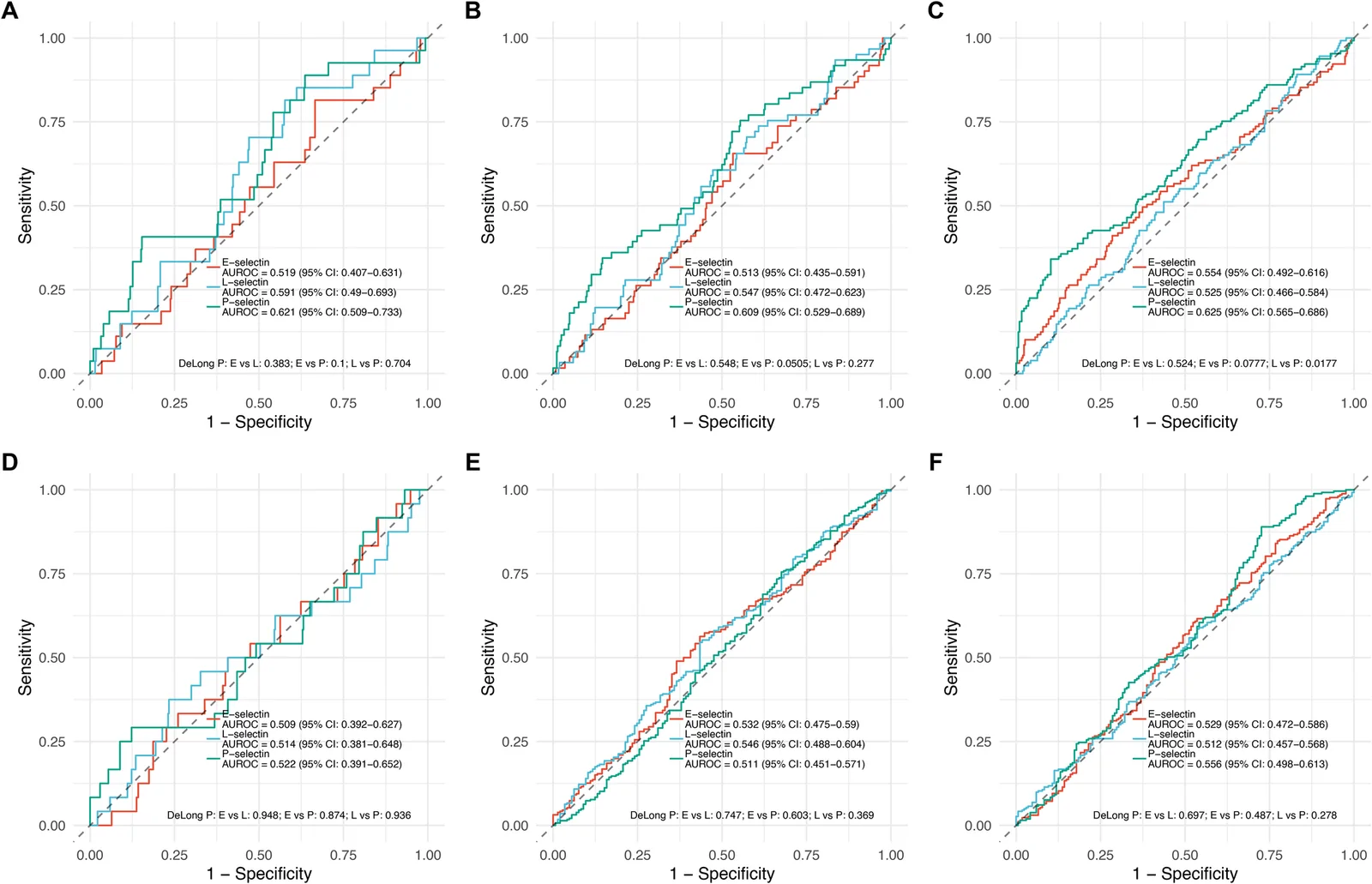
Receiver operating characteristic (ROC) curves evaluating the predictive performance of serum selectins in the full unmatched cohort. **(A–C)** ROC curves predicting overall survival at 3 months, 12 months, and 3 years in the full unmatched cohort. **(D–F)** ROC curves predicting independent ambulation at 3 months, 12 months, and 3 years in the full unmatched cohort. The area under the ROC curve (AUROC) with 95% confidence intervals and DeLong test *p* values for pairwise comparisons between markers are shown within each panel. ROC analyses in the matched cohort and time-dependent ROC analyses are presented in Supplementary Figures 2 and 3, respectively.

In the full unmatched cohort (Figures 2A–F), the area under the ROC curve (AUROC) was calculated to assess predictive accuracy. For overall survival (Figures 2A–C), P-selectin showed the highest discrimination, with AUROC values of 0.621 (95% CI: 0.503–0.733) at 3 months, 0.609 (0.521–0.688) at 12 months and 0.625 (0.565–0.686) at 3 years, compared with 0.511–0.554 for E-selectin and 0.525–0.591 for L-selectin. For the prediction of independent ambulation (Figures 2D–F), discrimination was close to chance for all three markers, with AUROC values ranging from 0.503 to 0.556 (E-selectin 0.503–0.532; L-selectin 0.512–0.548; P-selectin 0.511–0.556); DeLong tests revealed no significant pairwise differences between markers (all *p* > 0.27).

ROC analyses were repeated in the 1:1 propensity-score-matched case-control subset (Supplementary Figure S2). P-selectin again showed the highest AUROC for survival at 3 months (0.648, 95% CI: 0.533–0.752), 12 months (0.627, 0.542–0.711) and 3 years (0.656, 0.585–0.726), whereas all three markers performed close to chance for independent ambulation (AUROC 0.487–0.548). Because the matched subset is a case-control sample in which the case fraction is fixed at 50% by design, these AUROCs are reported as exploratory only and are not interpretable as properties of a general hip-fracture population. Time-dependent ROC analyses that formally account for censoring are presented in Supplementary Figure S3; in the unmatched cohort, P-selectin yielded AUROC values of 0.638 at 3 months, 0.632 at 12 months and 0.656 at 3 years, while in the matched cohort the corresponding values were 0.655, 0.613 and 0.528. To facilitate clinical application and risk stratification, the optimal threshold for each biomarker was determined from the ROC curve for 3-year overall survival in the full unmatched cohort by maximising the Youden index, which optimises the balance between sensitivity and specificity. The resulting cutoffs were 413.39 pg/mL for E-selectin, 17.17 ng/mL for L-selectin, and 16.64 ng/mL for P-selectin. Using these thresholds, patients were dichotomised into a below-cutoff group (values at or below the cutoff) and an above-cutoff group (values above the cutoff) for each marker. These strata are study-derived and do not imply a clinical reference range; this categorisation allowed further targeted analysis of the impact of elevated biomarker expression on post-operative outcomes.

### Clinical outcomes

To further investigate the prognostic significance of the dichotomised selectins, Kaplan–Meier survival analyses were conducted over the 36-month follow-up period. Differences between the below-cutoff and above-cutoff groups were formally assessed using the log-rank test. As depicted in the survival curves (Figures 3A–F), patients with above-cutoff E-selectin and above-cutoff P-selectin levels demonstrated significantly lower survival probabilities than those in the below-cutoff groups (log-rank *p* = 0.011 and *p* < 0.0001, respectively), whereas L-selectin did not significantly stratify overall survival (log-rank *p* = 0.12). In the matched cohort (Figures 3G–L), the same pattern was observed, with a significant difference for P-selectin (log-rank *p* < 0.0001), a borderline difference for E-selectin (log-rank *p* = 0.054) and no significant stratification for L-selectin (log-rank *p* = 0.21). Numbers at risk and 95% confidence bands are shown in Figure 3.

**Figure 3:**
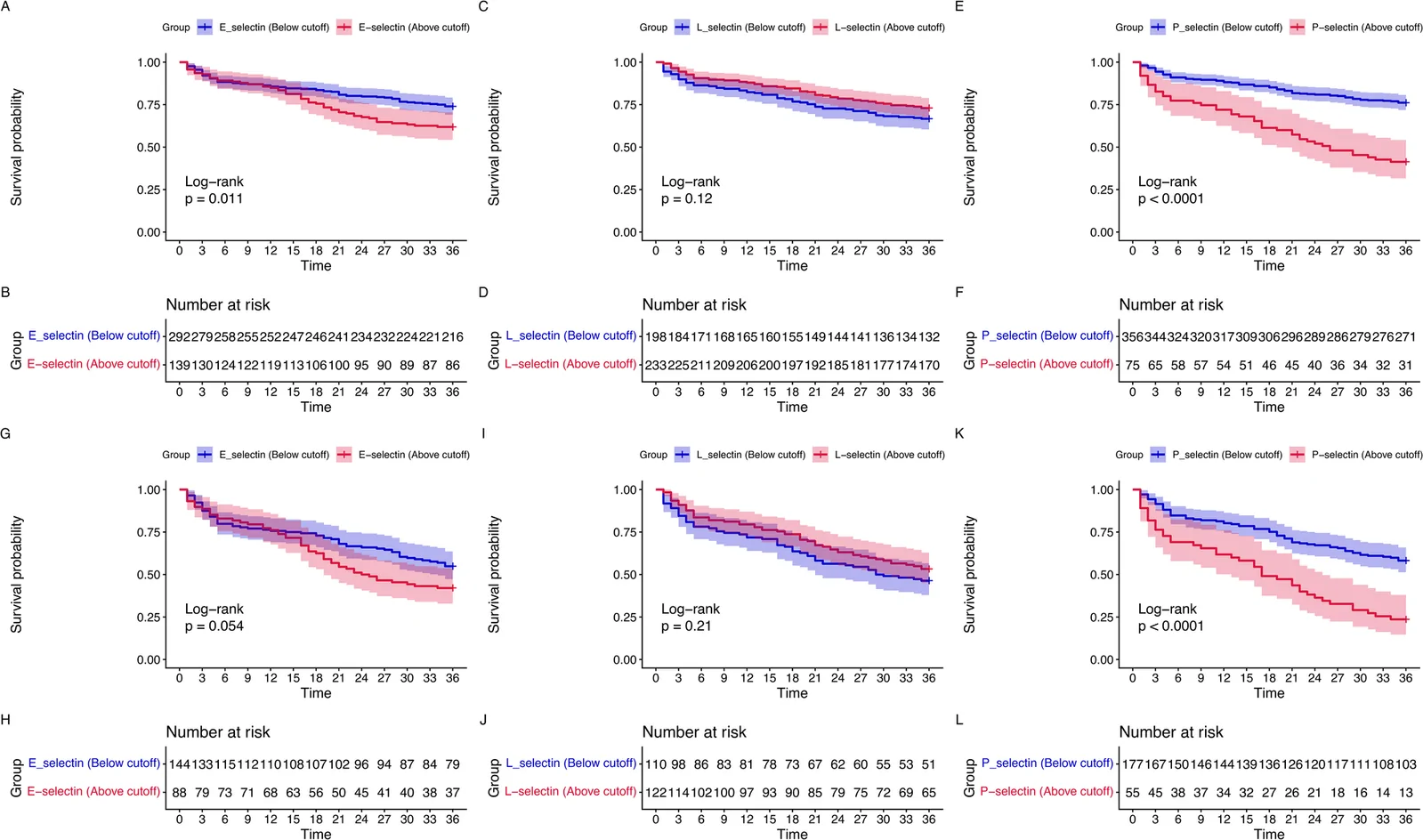
Kaplan–Meier survival analysis stratified by baseline serum selectin levels, with numbers at risk shown beneath each curve. In the full unmatched cohort, patients were dichotomised into below-cutoff and above-cutoff groups using the optimal cutoff values for E-selectin **(A,B)**, L-selectin **(C,D)**, and P-selectin **(E,F)**. Panels G-L show the corresponding analyses in the 1:1 propensity-score-matched cohort for E-selectin **(G,H)**, L-selectin **(I,J)** and P-selectin **(K,L)**. Shaded regions represent 95% confidence intervals, and *p* values are from the log-rank test.

To provide granular insight into functional and survival endpoints, mortality and independent ambulation rates at 3 months, 12 months and 3 years were compared across the dichotomised biomarker groups in the full cohort (Table 2), in which all 431 patients contributed to the denominator; patients who had died were counted as not having regained independent ambulation. Patients in the above-cutoff E-selectin group (*n* = 139) experienced a significantly higher 3-year mortality rate than the below-cutoff group (*n* = 292) (38.13% vs. 26.03%; RR = 1.46, 95% CI: 1.10–1.95; *p* = 0.01), whereas no statistically significant differences were observed regarding 3-month or 12-month mortality, or independent ambulation at any time point (all *p* > 0.05). Stratification by L-selectin thresholds (below cutoff *n* = 198; above cutoff *n* = 233) yielded no statistically significant differences across any of the recorded clinical endpoints, with short-term and long-term mortality, as well as postoperative independent ambulation rates, remaining comparable between the two groups (all *p* > 0.05). In contrast, above-cutoff P-selectin (*n* = 75) identified a group with markedly worse clinical outcomes than the below-cutoff group (*n* = 356), with significantly elevated mortality at 3 months (14.67% vs. 4.49%; RR = 3.26, 95% CI: 1.58–6.75; *p* = 0.002), 12 months (28.00% vs. 11.24%; RR = 2.49, 95% CI: 1.56–3.97; *p* < 0.001) and 3 years (58.67% vs. 23.88%; RR = 2.46, 95% CI: 1.88–3.20; *p* < 0.001), and a substantially lower 3-year independent ambulation rate (38.67% vs. 65.73%; RR = 0.59, 95% CI: 0.44–0.79; *p* < 0.001). Differences in earlier functional recovery did not reach statistical significance (3-month independent ambulation *p* = 0.708; 12-month *p* = 0.121). Because death precludes the observation of ambulation, these full-cohort rates represent a composite of death or dependence. We therefore repeated the analysis in the 302 patients who survived to 3 years (Supplementary Table S3): within this survivor population, 263 of 302 patients (87.09%) had regained independent ambulation at 3 years, and the difference between the below-cutoff and above-cutoff P-selectin groups was no longer statistically significant (86.35% vs. 93.55%, *p* = 0.395), indicating that the apparent association between P-selectin and ambulation in the full cohort is largely attributable to the competing risk of death.

**Table 2:** Comparison of clinical outcomes across dichotomised serum selectin groups at 3 months, 12 months, and 3 years in the full cohort.

Outcomes	E-selectin (below cutoff)	E-selectin (above cutoff)	Risk ratios (95% CI)	*p* value
	(*n* = 292)	(*n* = 139)		
3-month mortality	17 (5.82%)	10 (7.19%)	1.24 (0.58–2.63)	0.583
12-month mortality	40 (13.70%)	21 (15.11%)	1.10 (0.68–1.80)	0.695
3-year mortality	76 (26.03%)	53 (38.13%)	1.46 (1.10–1.95)	0.01
3-month independent ambulation	16 (5.48%)	8 (5.76%)	1.05 (0.46–2.40)	0.907
12-month independent ambulation	196 (67.12%)	90 (64.75%)	0.96 (0.83–1.12)	0.626
3-year independent ambulation	185 (63.36%)	78 (56.12%)	0.89 (0.75–1.05)	0.15

Outcomes	L-selectin (below cutoff)	L-selectin (above cutoff)	Risk ratios (95% CI)	*p* value
	(*n* = 198)	(*n* = 233)		
3-month mortality	17 (8.59%)	10 (4.29%)	0.50 (0.23–1.07)	0.067
12-month mortality	34 (17.17%)	27 (11.59%)	0.67 (0.42–1.08)	0.097
3-year mortality	66 (33.33%)	63 (27.04%)	0.81 (0.61–1.08)	0.155
3-month independent ambulation	12 (6.06%)	12 (5.15%)	0.85 (0.39–1.85)	0.681
12-month independent ambulation	122 (61.62%)	164 (70.39%)	1.14 (1.00–1.31)	0.055
3-year independent ambulation	116 (58.59%)	147 (63.09%)	1.08 (0.92–1.25)	0.339

Outcomes	P-selectin (below cutoff)	P-selectin (above cutoff)	Risk ratios (95% CI)	*p* value
	(*n* = 356)	(*n* = 75)		
3-month mortality	16 (4.49%)	11 (14.67%)	3.26 (1.58–6.75)	0.002
12-month mortality	40 (11.24%)	21 (28.00%)	2.49 (1.56–3.97)	<0.001
3-year mortality	85 (23.88%)	44 (58.67%)	2.46 (1.88–3.20)	<0.001
3-month independent ambulation	21 (5.90%)	3 (4.00%)	0.68 (0.21–2.22)	0.708
12-month independent ambulation	242 (67.98%)	44 (58.67%)	0.86 (0.70–1.06)	0.121
3-year independent ambulation	234 (65.73%)	29 (38.67%)	0.59 (0.44–0.79)	<0.001

### Multivariate analyses

To determine the independent prognostic value of the selectins, univariable and multivariable regression analyses were conducted for both survival and functional recovery outcomes. As detailed in Table 3 and Supplementary Table S4, Cox proportional hazards models were used to evaluate overall survival. In the univariable analyses, both the continuous and the categorised terms for E-selectin and P-selectin were significantly associated with an increased risk of mortality, whereas L-selectin showed no prognostic significance (*p* > 0.05). Two multivariable Cox models were prespecified. Model 1 was adjusted for age and time from injury to surgery, the only variables that differed between groups on univariable screening; above-cutoff P-selectin remained a robust independent risk factor for mortality (HR = 2.967, 95% CI: 2.056–4.280, *p* < 0.001), whereas above-cutoff E-selectin showed only borderline significance (HR = 1.416, 95% CI: 0.995–2.015, *p* = 0.053). Model 2 was adjusted for a broader, clinically motivated set of covariates (age, sex, CCI, BMI, hemoglobin, albumin, platelet count, CRP, fracture location, type of surgical procedure and time from injury to surgery). In this fully adjusted model, above-cutoff P-selectin remained the strongest independent predictor of 3-year mortality (HR = 3.167, 95% CI: 2.170–4.622, *p* < 0.001), whereas the association with above-cutoff E-selectin was attenuated and no longer statistically significant (HR = 1.397, 95% CI: 0.971–2.011, *p* = 0.072). The same pattern was observed for the continuous terms: P-selectin remained independently associated with mortality in both models (Model 2: HR = 1.050 per ng/mL, 95% CI: 1.029–1.071, *p* < 0.001), whereas E-selectin did not (Model 2: HR = 1.007 per pg/mL, 95% CI: 0.999–1.014, *p* = 0.084). The proportional hazards assumption was satisfied for every model (global Schoenfeld-residual test *p* > 0.5 for both multivariable models), and the numbers of patients, events and events per variable are reported in Supplementary Table S5.

**Table 3:** Univariable and multivariable cox proportional hazards regression analyses for predictors of 3-year all-cause mortality.

Variables	Univariate Model		Multivariate Model 1		Multivariate Model 2	
	HR (95% CI)	*p*	HR (95% CI)	*p*	HR (95% CI)	*p*
E-selectin (continuous)	1.009 [1.001, 1.016]	0.022	1.007 [1.000, 1.015]	0.051	1.007 [0.999, 1.014]	0.084
L-selectin (continuous)	0.989 [0.968, 1.011]	0.318	0.990 [0.969, 1.012]	0.361	0.988 [0.966, 1.010]	0.283
P-selectin (continuous)	1.054 [1.033, 1.075]	<0.001	1.048 [1.028, 1.069]	<0.001	1.050 [1.029, 1.071]	<0.001
E-selectin (above cutoff)	1.569 [1.105, 2.229]	0.012	1.416 [0.995, 2.015]	0.053	1.397 [0.971, 2.011]	0.072
L-selectin (Above cutoff)	0.760 [0.538, 1.074]	0.12	0.785 [0.556, 1.108]	0.169	0.735 [0.516, 1.047]	0.088
P-selectin (above cutoff)	3.172 [2.202, 4.570]	<0.001	2.967 [2.056, 4.280]	<0.001	3.167 [2.170, 4.622]	<0.001

Continuous selectin terms are expressed per 10 pg/mL for E-selectin and per 1 ng/mL for L- and P-selectin.

Logistic regression models were employed to identify independent predictors of independent ambulation (Table 4 and Supplementary Tables S6, S7). Consistent with the survival analyses, L-selectin and E-selectin were not significantly associated with functional recovery at 3 months, 12 months or 3 years in the multivariable models. Above-cutoff P-selectin was, however, a significant independent predictor of a reduced likelihood of achieving independent ambulation at 3 years in the full cohort, both in Model 1 (OR = 0.357, 95% CI: 0.209–0.600, *p* < 0.001) and in Model 2 (OR = 0.346, 95% CI: 0.201–0.587, *p* < 0.001). Because ambulation can only be observed in survivors, these models were repeated in the 302 patients who survived to 3 years (Supplementary Table S7). In this survivor-restricted analysis the dichotomised P-selectin term was no longer statistically significant (Model 2: OR = 2.393, 95% CI: 0.663–15.400, *p* = 0.253), and the continuous P-selectin term showed only a weak association that was inconsistent in direction with the full-cohort estimate (Model 2: OR = 1.104 per ng/mL, 95% CI: 1.028–1.210, *p* = 0.015), with a wide confidence interval reflecting the small number of above-cutoff patients who survived (*n* = 31). Taken together, these findings indicate that the association between P-selectin and functional recovery in the full cohort is substantially driven by the competing risk of death.

**Table 4:** Multivariable logistic regression analyses identifying independent predictors of independent ambulation at 3, 12 and 36 months in the full cohort.

Variables	3-month independent ambulation		12-month independent ambulation		3-year independent ambulation	
	OR (95% CI)	*p*	OR (95% CI)	*p*	OR (95% CI)	*p*
Multivariate Model 1						
E-selectin (continuous)	0.996 [0.977, 1.014]	0.655	0.995 [0.986, 1.003]	0.215	0.993 [0.985, 1.002]	0.13
L-selectin (continuous)	0.994 [0.944, 1.048]	0.825	1.020 [0.995, 1.047]	0.12	1.006 [0.981, 1.031]	0.664
P-selectin (continuous)	1.001 [0.947, 1.051]	0.972	0.986 [0.961, 1.011]	0.254	0.970 [0.946, 0.994]	0.015
E-selectin (above cutoff)	1.021 [0.403, 2.395]	0.963	0.943 [0.615, 1.455]	0.79	0.798 [0.524, 1.219]	0.295
L-selectin (above cutoff)	0.852 [0.370, 1.964]	0.704	1.458 [0.974, 2.187]	0.067	1.173 [0.788, 1.746]	0.432
P-selectin (above cutoff)	0.630 [0.145, 1.908]	0.467	0.713 [0.427, 1.203]	0.2	0.357 [0.209, 0.600]	<0.001
Multivariate Model 2						
E-selectin (continuous)	0.994 [0.975, 1.013]	0.556	0.995 [0.986, 1.003]	0.232	0.993 [0.985, 1.002]	0.138
L-selectin (continuous)	0.985 [0.934, 1.039]	0.573	1.022 [0.995, 1.049]	0.106	1.005 [0.979, 1.031]	0.708
P-selectin (continuous)	1.002 [0.947, 1.054]	0.94	0.985 [0.960, 1.011]	0.258	0.967 [0.943, 0.992]	0.01
E-selectin (above cutoff)	0.946 [0.364, 2.275]	0.904	0.932 [0.602, 1.453]	0.755	0.785 [0.510, 1.210]	0.271
L-selectin (above cutoff)	0.722 [0.302, 1.713]	0.457	1.489 [0.986, 2.253]	0.059	1.179 [0.786, 1.769]	0.426
P-selectin (above cutoff)	0.609 [0.138, 1.884]	0.441	0.700 [0.415, 1.192]	0.185	0.346 [0.201, 0.587]	<0.001

Continuous selectin terms are expressed per 10 pg/mL for E-selectin and per 1 ng/mL for L- and P-selectin.

## Discussion

This 3-year, single-center, observational cohort study demonstrates that an elevated preoperative serum P-selectin concentration is an independent predictor of long-term all-cause mortality in older adults undergoing hip fracture surgery. Specifically, high baseline P-selectin emerged as a highly robust indicator of poor clinical outcomes, associating significantly with an increased risk of mortality and a drastically reduced likelihood of restoring independent ambulation. Multivariable Cox and logistic regression in the full cohort confirmed above-cutoff P-selectin as an independent predictor of both 3-year mortality and functional decline, whereas the association between E-selectin and mortality did not persist after adjustment. In a 1:1 propensity-score-matched case-control subset in which baseline clinical, demographic and laboratory covariates were well balanced, P-selectin levels remained significantly higher in patients who died within 3 years than in survivors. Conversely, while L-selectin is a recognized mediator of leukocyte rolling, it demonstrated no prognostic utility for either overall survival or the recovery of independent living ability in this cohort.

The mechanistic interpretation that follows is hypothesis-generating, because none of the pathways described below was measured directly in this study. The pronounced predictive value of P-selectin for both mortality and functional impairment likely reflects the molecule's central role in initiating and amplifying thromboinflammation. P-selectin is prestored in the α-granules of platelets and the Weibel-Palade bodies of endothelial cells, from which it is rapidly translocated to the cell surface upon activation (17). Surgical intervention for osteoporotic fractures induces a state of systemic inflammation. In this context, platelet P-selectin interacts with monocyte P-selectin glycoprotein ligand-1 (PSGL-1) to form platelet-monocyte aggregates (17). These aggregates may exert profound inflammatory effects, contributing to vascular dysfunction, microvascular thrombosis, and systemic immune dysregulation (29–32).

Furthermore, high concentrations of P-selectin have been positively correlated with the incidence of sarcopenia. Because sarcopenia inherently impairs muscle mass and postural stability, the systemic inflammatory burden heralded by elevated P-selectin is closely linked to the compromised long-term functional recovery and independent ambulation observed in our above-cutoff P-selectin group (25, 33). Moreover, P-selectin enhances fibrin deposition and serves as a key molecule in the pathogenesis of venous thromboembolism, a severe and frequent complication following major orthopedic surgery in the older adults (14, 34). The combination of heightened thrombosis risk and chronic inflammation provides a strong mechanistic rationale for the 3.17-fold increased hazard of 3-year mortality observed in patients with above-cutoff P-selectin.

In the univariable analysis, elevated E-selectin was also associated with poorer survival, but this association was attenuated and no longer statistically significant after multivariable adjustment, and it should therefore be regarded as exploratory. Unlike P-selectin, E-selectin is exclusively expressed on endothelial cells, and its expression is not constitutive but is strongly induced by pro-inflammatory cytokines and oxidative stress that trigger endothelial cell injury (10, 20). The univariable association between preoperative E-selectin and 3-year mortality is consistent with a contribution of endothelial activation and vascular derangement at admission to adverse long-term surgical outcomes; however, given the fragility of this signal (univariable *p* = 0.012; adjusted *p* = 0.072; absolute AUROC no greater than 0.556) and the multiplicity of markers, parameterisations and time points examined, this finding requires confirmation in larger, adequately powered cohorts before it can be considered clinically useful.

L-selectin is primarily expressed on circulating leukocytes and is essential for their initial tethering to the vascular endothelium during inflammatory cascades (11, 18, 19, 35). Despite its physiological importance, L-selectin levels failed to stratify risk for either mortality or ambulation in our cohort. This finding suggests that while leukocyte recruitment is a component of the post-fracture inflammatory response, the primary drivers of adverse long-term outcomes in this population may be potentially rooted in platelet and endothelial activation.

It is important to note that while P-selectin was identified as a significant independent risk factor in our multivariable analyses, its absolute predictive accuracy as a standalone marker was modest, with AUROC values peaking at 0.625 in the full unmatched cohort and 0.656 in the time-dependent ROC analysis. This limited discriminative ability indicates that P-selectin should not be utilized in isolation for clinical decision-making. Instead, its optimal clinical utility likely resides in its integration with established clinical parameters, such as patient age, the Charlson Comorbidity Index, and other systemic inflammatory indices, to develop a comprehensive, multi-variable prognostic risk score. Future studies utilizing larger, multi-center cohorts are warranted to construct and externally validate such combined risk models, which could significantly enhance predictive accuracy and better guide personalized interventions for older adults recovering from hip fractures.

The final cohort of 431 patients yielded 129 deaths during the 3-year follow-up period. With 129 events, the fully adjusted model containing 12 parameters had approximately 10.8 events per variable, which is close to the conventional minimum of 10 and therefore limits the precision of the individual covariate estimates; this is reflected in the width of the confidence interval for the P-selectin hazard ratio, which spans 2.170–4.622. Because the observed event count is fixed by the data, this precision-based description is more informative than a *post-hoc* power calculation, which would be circular.

However, several limitations must be acknowledged. First, the single-center, observational design inherently limits the generalizability of the findings and precludes the establishment of direct causality. Second, the primary outcome was all-cause mortality and the exact causes of death were not categorized; given the established roles of selectins in thrombosis and systemic inflammation, distinguishing cardiovascular from non-cardiovascular deaths would provide important mechanistic insight. Third, selectin concentrations were measured only once, on the first morning after admission, and therefore do not capture the dynamic postoperative trajectory of inflammation and selectin expression; the interval from injury to admission was also not recorded. Fourth, the cutoffs used for stratification were derived by maximising the Youden index and then applied within the same dataset, which may inflate effect estimates; the continuous biomarker models are therefore presented as the primary analyses (Tables 3, 4) and the dichotomised analyses as secondary, exploratory analyses. Fifth, the P-selectin cutoff of 16.64 ng/mL identified a relatively small group (*n* = 75 of 431, 17.4%), which limits the stability of the corresponding effect estimates, and the matched case-control comparison was based on a moderate sample size (232 patients). Sixth, because death precludes the observation of ambulation, the full-cohort ambulation analyses represent a composite of death or dependence; when the analysis was restricted to the 302 patients who survived to 3 years, the association between P-selectin and independent ambulation was no longer statistically significant (Supplementary Tables S3 and S7), and future studies should use a competing-risks framework such as the Fine-Gray subdistribution model. Seventh, we did not evaluate pre-fracture ambulatory status or cognitive function; because these are among the strongest known predictors of postoperative ambulation, their absence from our multivariable models is an important limitation, and our findings regarding functional recovery should therefore be interpreted with caution. Eighth, ambulation was ascertained using a locally defined binary criterion rather than a validated instrument such as the New Mobility Score or the Functional Ambulation Category, and we did not record medication history that directly affects soluble selectins, including antiplatelet agents, anticoagulants and statins. Ninth, our study utilized serum rather than citrated plasma for the quantification of selectins; consequently, our measured serum values are inherently higher than true *in vivo* circulating levels, and direct comparisons with plasma-based studies are limited. Tenth, because of variations across different analytical platforms, our specific numerical cutoffs are platform-dependent and should not be extrapolated to cohorts assayed with other commercial kits. In addition, we did not formally quantify the incremental prognostic value of P-selectin beyond routine clinical and laboratory predictors, and this remains to be addressed in future prospective, externally validated studies. We also did not compare the baseline characteristics of the 96 screened patients who were excluded because no valid serum selectin measurement was available, or of the 42 patients lost to follow-up, with those of the analysed cohort, so selection bias cannot be excluded.

## Conclusions

Elevated preoperative serum P-selectin is an independent predictor of long-term mortality in older adults following hip fracture surgery. Above-cutoff P-selectin was also associated with a reduced likelihood of regaining independent ambulation, although this association was attenuated when the analysis was restricted to 3-year survivors and is therefore best interpreted in the context of the competing risk of death. E-selectin showed a univariable association with mortality that did not persist after multivariable adjustment. Taken together, these findings are consistent with a role for platelet-mediated thromboinflammation in postoperative recovery and support the potential of P-selectin as a biomarker for clinical risk stratification in orthopedic trauma, pending external validation in larger, multicentre cohorts.

## Data Availability

The raw data supporting the conclusions of this article are not publicly available due to privacy concerns. Requests to access the datasets should be directed to the corresponding author.
