# Supplementary material for: Association of serum selectins with outcomes in older adults following hip fracture surgery: a 3-year follow-up study
Source: Front Med (Lausanne). 2026 Oct 27;13:1932543. doi: 10.3389/fmed.2026.1932543 (PMC13747958; doi:10.3389/fmed.2026.1932543)
Supplement: Supplementary file 1 [file Datasheet1.docx]

Supplementary Figure 1: Love Plot evaluating the covariate balance before and after matching.

Supplementary Figure 2. Receiver Operating Characteristic (ROC) curves evaluating the predictive performance of serum selectins.

(A–C) ROC curves predicting survival outcomes at 3 months, 12 months, and 3 years following 1:1 propensity score matching (PSM). (D–F) ROC curves predicting independent ambulation at 3 months, 12 months, and 3 years in the PSM cohort.

Supplementary Figure 3. Time-dependent Receiver Operating Characteristic (ROC) curves evaluating the predictive performance of serum selectins.

(A–C) Time-dependent ROC curves predicting overall survival at 3 months, 12 months, and 3 years in the unmatched cohort. (D–F) Time-dependent curves predicting survival outcomes at 3 months, 12 months, and 3 years following 1:1 propensity score matching (PSM).
